# Directionality of information flow and echoes without chambers

**DOI:** 10.1371/journal.pone.0215949

**Published:** 2019-05-15

**Authors:** Soojong Kim

**Affiliations:** Annenberg School for Communication, University of Pennsylvania, Philadelphia, Pennsylvania, United States of America; Spanish National Research Council, SPAIN

## Abstract

How do echo chambers operate? Why does social propagation of information become trapped within the boundaries of social groups? Previous studies of these questions have identified informational and structural factors which hinder information exchange across group boundaries; these factors constitute “chambers” in which information flows are confined and transformed into “echoes.” However, empirical evidence has indicated that these factors may not sufficiently explain the mechanism of echo chambers. Hence, the present study investigated whether the insular flow of information emerges and endures without the chambers. A randomized controlled experiment was conducted in which participants, who were classified into two political groups, exchanged randomly selected articles with the same number of ingroup and outgroup neighbors. The experiment manipulated the directionality of incoming information flow by varying the number of articles sent from ingroup neighbors across two conditions. Analyses revealed that the ingroup-slanted inflow induced ingroup-slanted outflow, suppressing transmission toward neighbors in a different social group. The biased inflow also promoted positive reactions to information exchanges and reduced negative evaluations on the exchanged information. Furthermore, the ingroup-slanted inflow increased false perceptions of ingroup majority, which is known to encourage information dissemination by a social group. The present study suggests two self-reinforcing mechanisms of ingroup-biased flows that generate echoes even without the chambers. These mechanisms may enable a small group of strategic actors to exacerbate polarization within a large population by manipulating directions of information flow.

## Introduction

Information spreads through pathways of social networks and reaches a large population within a short period of time [1–3]. The Internet and social media have reduced temporal and spatial constraints on communication, further facilitating information dissemination on social networks. However, social propagation of information is still often constrained by people’s social identities, such as race, gender, and political affiliation. The spread of information tends to be bounded within a social group, leading to “echo chambers” that could cause social segregation and political polarization [4–7].

Why do the boundaries of social groups hinder the propagation of information? How do echo chambers form and operate? Previous attempts to answer these questions have mainly focused on two types of segregation that can induce an insular pattern of information flow. Some scholars have explained that people are selectively exposed to information that supports their viewpoints. Due to cognitive biases [8–10], people prefer information that confirms their existing beliefs and avoid uncongenial information [7,11–16]. According to this approach, information that supports the beliefs of a social group tends to be circulated within the group. For example, articles that favor conservative standpoints are more likely to be transmitted from and received by conservatives [4,17]. Thus, the topics and the content of information determine its scope of propagation, and this phenomenon can be called “informational segregation.” Another group of scholars has found that cross-cutting communication on social networks is structurally inhibited [5,6,18–20]. Social ties tend to connect people in the same social group [21], and, due to this “structural segregation,” ingroup interaction occurs at a higher rate than outgroup interaction. Research based on this approach has focused on the measurement of structural homophily to detect the presence of echo chambers [5,6,18].

However, empirical evidence has indicated that these factors may not sufficiently explain the mechanisms underpinning echo chambers. First, echo chambers and ingroup-biased behaviors have been observed even when information is hardly relevant to the beliefs of social groups [4,22]. Also, increases in the amount of information in the current media environment have made it difficult to thoroughly examine topics and content of information [23,24], and evaluation of content often ends up revealing ambiguity of information in relation to opinions of social groups [25]. Second, it has been found that individuals on social media not only maintain a considerable number of cross-cutting social connections [6] but also seek exposure to content supporting the opinions of an opponent group [4,26,27]. These pieces of evidence indicate that there may be an undiscovered dimension of the mechanism and dynamics of echo chambers.

The current study focused on an overlooked mechanism of echo chambers considering social motivations for information exchanges. Because of the desire for positive social relations [28], information received from ingroup members may motivate people to share it with other ingroup members. I examined whether the insular flow of information emerges and endures due to this “inertia” of flows, even without informational or structural segregation. I first defined ingroup directionality of incoming (outgoing) information flow as the proportion of information that an individual receives from (transmits to) his/her ingroup members. The present study investigated the effects of information flows by comparing two different levels of ingroup directionality of inflow. First, “ingroup-biased” inflow refers to a condition in which the majority of information comes from a person’s ingroup neighbors. Second, “balanced” inflow refers to a condition in which the same amount of information comes from ingroup and outgroup neighbors. (Outgroup-biased inflow, which refers to a condition in which the majority of information comes from a person’s outgroup neighbors, was not investigated in the present study.)

The present study tested three hypotheses. First, it hypothesized that the ingroup-biased inflow increases ingroup directionality of outgoing flow. Since the increased ingroup directionality of outflow consecutively increases the ingroup directionality of inflow for other individuals, the hypothesized effect could create cascades in social networks, resulting in “echoes” that reverberate within a social group. The second hypothesis is that the ingroup-biased inflow promotes a false perception of an ingroup majority. Because the perceived majority of ingroup encourages the group’s information dissemination and amplifies flows from the group toward other individuals [29,30], this hypothetical effect could also induce ripple effects on social networks and contribute to the separation of information flows. Lastly, the current study hypothesized that the ingroup-biased inflow promotes positive reactions to social interactions and positive perceptions of shared information. If the biased flow results in better social relationships and emotional states, people may be motivated to maintain and reproduce the insular flow of information.

To test these hypotheses, I carried out an experiment in which participants from two political groups exchanged articles with their neighbors. Participants received randomly selected articles from the same number of ingroup and outgroup neighbors. They also transmitted the articles to neighbors of their choosing. In this environment, I manipulated ingroup directionality of inflow by varying the number of articles sent from ingroup members across two conditions.

### Social motivation for information exchange

The social propagation of information is made possible by cascades of certain behaviors, receiving information from and transmitting information to others on social networks. While contents and social structures affect how information propagates on social networks, people also rely on social cues during information exchange [31–33]. One such cue that people use to exchange information is the social identity of sending and receiving neighbors. A preference for ingroup members and the expectation of favorable interaction with them can shape social behaviors during information propagation [28,34–36].

Specifically, processes of ingroup favoritism may influence the direction of transmission. First, information sent by an ingroup member may inform the receiving individual that the ingroup sender had evaluated the information as favorable for their social group. Thus, stimulating the individual’s ingroup bias to benefit other ingroup members, the ingroup reception of information triggers its ingroup transmission. Second, an important, but largely overlooked, factor in the literature on information exchange is that people presume and expect others’ ingroup favoritism. Positive and pleasant social contact is crucial for satisfying the need to belong, building trust, and earning reputation, and people presume that positive interactions are more likely with ingroup members than outgroup ones [28,36,37]. Hence, signaling the likelihood of favorable interaction, the social identity of potential receivers may affect decisions about transmission direction. The effects of the expected ingroup favoritism could be stronger with ambiguous information or conflicting social cues: forwarding information to ingroup members, in this case, could be a strategic way to reduce the risk of negative social interactions. These explanations suggest that if information inflow is dominated by ingroup neighbors, it is likely to increase the ingroup directionality of outflow, promoting positive reactions during social interactions and positive perceptions of shared information.

Moreover, a slanted inflow may cause a distorted perception of social networks. Scholars have identified that estimation of social networks is subjective and inaccurate: a person’s perception of social structure is correlated with how he/she interacts with others [38,39]. Since ingroup-slanted inflow induces frequent ingroup interaction, it may also promote the overestimation of ingroup members on social networks. Because individuals who feel that they belong to the majority are known to disseminate information more actively [29,30], a false perception of the majority could reinforce biased flows toward other individuals.

Therefore, the present study aimed to examine three hypotheses. First, it hypothesized that the ingroup-biased inflow of information induces ingroup-biased outflow. Second, it was hypothesized that the ingroup-biased inflow increases the likelihood that participants falsely perceive an ingroup majority. Lastly, the present study hypothesized that the ingroup-biased inflow increases positive reactions during social interactions and positive perceptions of shared information.

### Experiment

Participants accessed an online experiment website using a hyperlink posted on the online labor market, Amazon Mechanical Turk (MTurk). Prior to the experiment, they identified themselves as Democrat or Republican. Participants who identified themselves as independent were excluded from analysis. They then entered an interactive experiment with 6 Democrat and 6 Republican neighbors. These neighbors were actually preprogrammed agents, but the participants were led to believe that they were interacting with real people.

**Fig 1:**
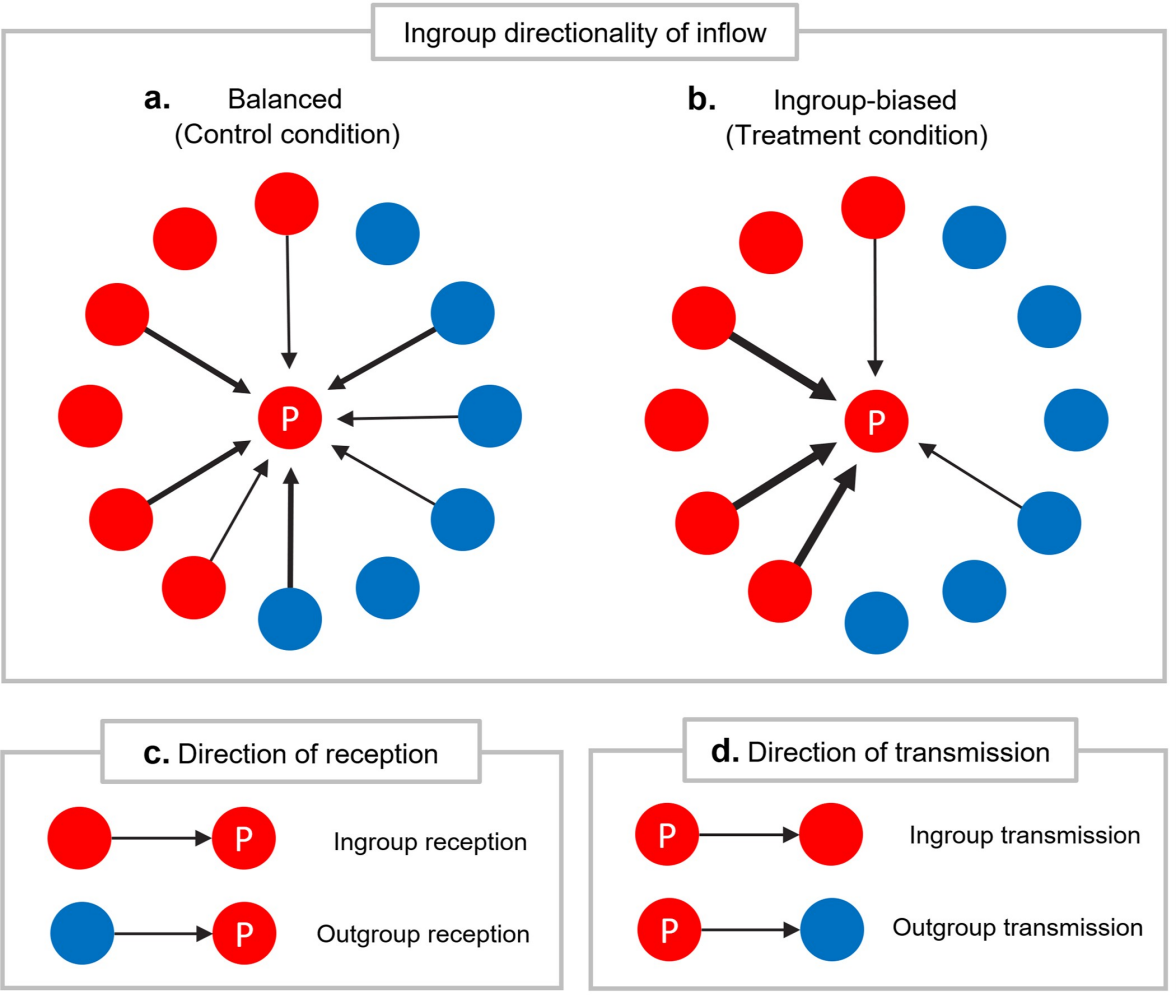
Experimental conditions and behavioral measures. Red (Republican) and blue (Democrat) colors depict the social identity of participants and neighbors, and these examples illustrate cases of Republican participants. A circle marked with “P” represents a participant, and a circle without a label represents a neighbor. An arrow connects a sender and a receiver of information. **a, b.** Ingroup directionality of inflow. Each participant was surrounded by 12 neighbors. The thickness of the arrows represents the relative amount of information received from neighbors. The total number of articles received from neighbors was the same in the two conditions. After receiving articles, the participants chose target neighbors for transmission. In the balanced condition, each group of neighbors sent the same number of articles to a participant (**a**). In the ingroup-biased condition, 11 of the 12 articles (92%) were sent from ingroup neighbors to a participant (**b**). **c**. Direction of reception. Ingroup[outgroup] reception refers to a case in which a participant receives an article from an ingroup[outgroup] neighbor. **d**. Direction of transmission. Ingroup[outgroup] transmission refers to a case in which a participant transmits an article to an ingroup[outgroup] neighbor.

The participants were randomly assigned to one of two conditions with different ingroup directionalities of inflow: a balanced inflow condition (Fig 1A) and an ingroup-biased inflow condition (Fig 1B). In the balanced inflow condition, each group of neighbors sent 6 articles to a participant, and a total of 12 articles were shared with participants. In the ingroup-biased condition, ingroup neighbors sent 11 articles among 12, creating an inflow with a high level of ingroup directionality (92%). Articles sent by neighbors were randomly chosen for each participant from a set of 42 articles, which described social and political issues in the United States (see Methods for details).

Participants repeated two phases during the experiment: the reception phase and the transmission phase. In the reception phase, participants were shown a list of articles. Articles were sequentially updated on the list displaying article titles and senders’ social identities. Once an article was selected, participants read and reacted to it. A click on a reaction button or an expiration of the reading timer initiated the transmission phase. In this phase, a participant chose a neighbor to transmit the selected article, and the chosen neighbor represented the social identity that the participant preferred the most as the target of a message transmission. They were informed that there would be a bonus or a penalty depending on whether the target neighbor would accept or reject their sharing. These incentives aimed to explicate and amplify participants’ consideration of social reactions in an experimental setting. Neighbors’ reactions were not disclosed to participants. After a target decision, participants returned to the reception phase and repeated the process for 6 minutes.

I measured variables related to information behaviors, information evaluation, and perceived network. Each time a participant opened an article from a neighbor, I checked ingroup reception, determining whether the neighbor was an ingroup member or an outgroup one (Fig 1C). A variable, positive reaction, represented whether the participant accepted the neighbor’s article sharing. When the participant chose a target neighbor for transmission, I examined ingroup transmission, whether or not the chosen neighbor was an ingroup member (Fig 1D). After the experiment, participants were asked three questions in which they identified articles that were supporting, challenging, and relevant to the opinions of their social groups, respectively. The perceived proportion of ingroup neighbors was measured after the experiment by asking participants to estimate what percentage of neighbors were from their own social group. See Methods for details.

The experimental design aimed to capture directional patterns of information exchange in social media. Although social media users can broadcast messages to all neighbors, they can also direct their information to specific individuals in various ways, for example, by referring to target receivers in messages, by sharing information with only a pre-specified group of neighbors, by including information in a thread of conversation, and by sending direct messages. Even when they send messages to all neighbors, they tend to prioritize and focus on a certain portion of neighbors as a target audience [17,40]. These directional patterns of information exchange in social media have been found to be crucial in understanding social segregation and political polarization [4,19,41].

The design offers important advantages over previous experimental attempts to investigate social propagation of information. First, participants interacted with human-like preprogrammed agents (bots). Compared with past studies using isolated environments that do not permit social interactions [42,43], this design allowed us not only to observe the social interactions of participants but also to maintain strict scientific controls. Second, participants exchanged information and reacted to neighbors in real time. The interactivity increases the ecological validity of the present study compared to previous studies based on static environments [11,43,44]. Third, this experiment encouraged participants to expect social feedback from neighbors. With this expectation, the experiment accommodated desires for reputation and interpersonal attachment, which are strong drivers of social interaction [28,36].

## Results

Participants selected 91.8% of the shared articles on average (M = 11.01, SD = 2.04), and 94.2% of them selected more than half of the shared articles. The participants read an article for 11.2 seconds (SD = 4.64) in the balanced condition and for 10.8 seconds (SD = 4.36) in the ingroup-biased condition on average. Those in the balanced and biased conditions selected 7.6 (SD = 1.62) and 7.3 (SD = 1.69) unique neighbors for transmission on average. For these observations, differences between the conditions were not statistically significant (see Methods for details). The results demonstrated that participants were able to distinguish each article based on their social identity and the message content, and a social group’s evaluation of an article was associated with the likelihood that the social group transmits the article to ingroup neighbors (see Methods for details).

**Fig 2:**
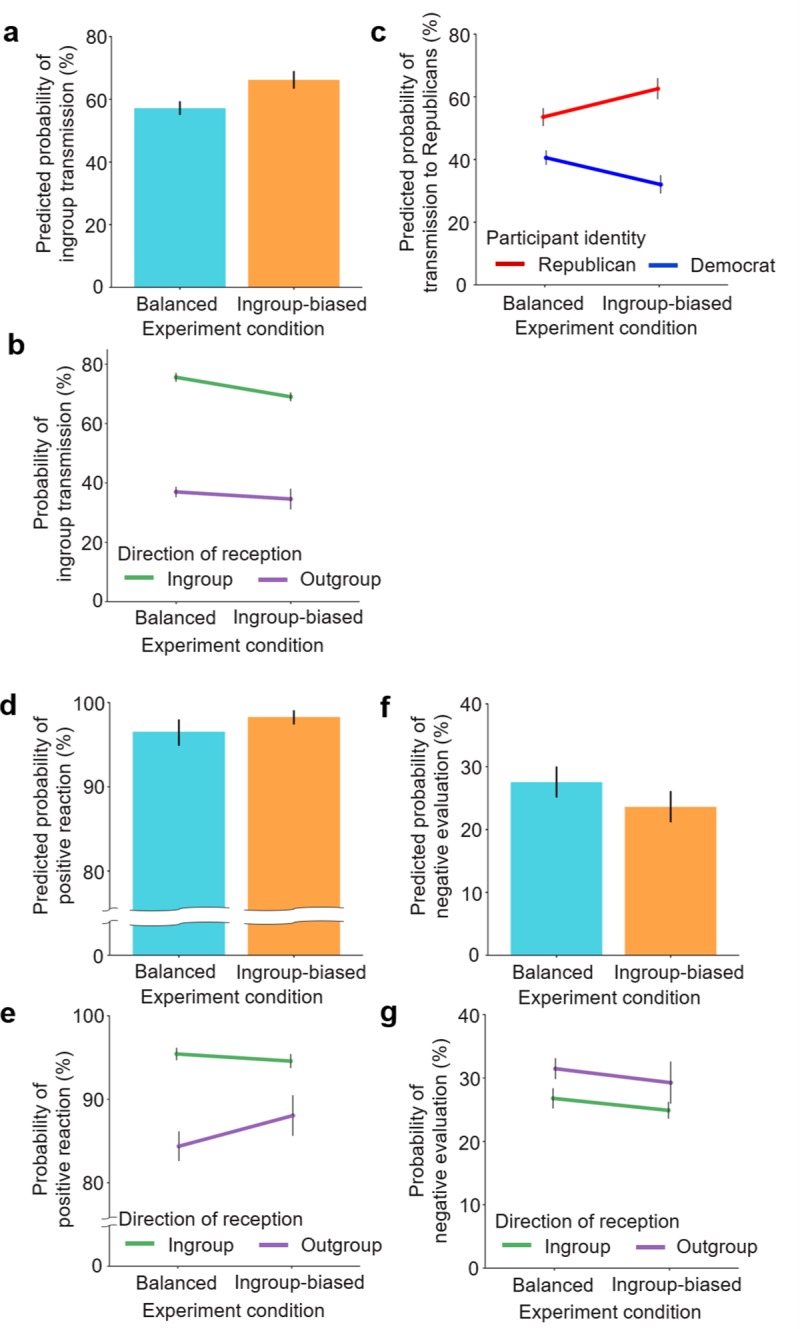
Behavioral-level effects of the ingroup-biased inflow on information behavior, social reaction, and information perception. *N* = 5,184 observations nested in 432 participants. (**a**, **c**, **d**, **f**) Predicted probability was calculated based on the model described in S4 Table. The error bars reflect 95% prediction intervals around the marginal means using bootstrapping of 1000 replicates. (**b**, **e**, **g**) The error bars reflect 95% confidence intervals around the means using bootstrapping of 1000 replicates. **a**. Predicted probability of ingroup transmission by condition. **b**. Predicted probability of transmission to Republicans by participant identity and condition. **c**. Probability of ingroup transmission by direction of reception and condition. **d**. Predicted probability of positive reaction by condition. **e**. Probability of positive reaction by direction of reception and condition. **f**. Predicted probability of negative evaluation by condition. **e**. Probability of negative evaluation by direction of reception and condition.

The ingroup-slanted inflow increased the probability of ingroup transmission by 9.1 percentage points from 57.1% in the balanced condition to 66.2% in the ingroup-biased condition (Fig 2A; *OR* = 1.45, 95% CI [1.27, 1.67], *P* = 1.29×10^−7^). The effect preserved the statistical significance in each subgroup, Democrat participants and Republican participants (see S2 Table for details). In terms of the probability of transmission toward Republicans, the difference between the two political groups of participants more than doubled, from 13.0% in the balanced condition to 30.6% in the biased condition, as shown in Fig 2C. Specifically, Republican and Democrat participants in the balanced condition transmitted 53.5% and 40.5% of the information they received to Republican neighbors, respectively, and the difference between the two groups was 13.0%. In the ingroup-biased condition, on the other hand, Republicans and Democrats transmitted 62.6% and 32.0% of their information to Republican neighbors, respectively, increasing the gap between the two groups to 30.6%.

The ingroup-slanted inflow also promoted positive social reactions (*OR* = 2.06, 95% CI [1.26, 3.42], *P* = 0.004) and suppressed negative evaluations of information (*OR* = 0.83, 95% CI [0.69, 0.99], *P* = 0.039). It increased the probability of positive reaction by 1.8 percentage points, from 96.5% in the balanced condition to 98.3% in the ingroup-biased one (Fig 2D), and reduced the probability of negative evaluation by 3.9 percentage points, from 27.5% to 23.6% (Fig 2F), although the effect sizes were small (Cohen’s *d* = 0.18 for both outcomes, see Methods for details). Statistically significant effects of the biased inflow were not detected for reading time, positive evaluation of information, and the evaluation of information relevancy. See S4 Table for details.

Ingroup reception was highly likely to be followed by ingroup transmission in both conditions. It was revealed that 75.6% (SE = 0.01%) and 69.0% (SE = 0.01%) of ingroup reception was accompanied with ingroup transmission in the balanced and the biased conditions, respectively, and these levels of association were much greater than those of outgroup reception, as shown in Fig 2B. A sizeable and highly significant correlation between ingroup reception and ingroup transmission was identified in both conditions (Balanced: *OR* = 5.56, 95% CI [4.67, 6.65], *P* = 9.02×10^−81^; Ingroup-biased: *OR* = 4.70, 95% CI [3.38, 6.58], *P* = 8.45×10^−20^; see S7 Table for details). In the balanced condition, the effect of ingroup reception preserved its statistical significance in both subgroups, Democrats and Republicans (S6 Table). Because the ingroup-slanted inflow is dominated by information coming from the ingroup, it could yield more frequent ingroup reception and ingroup transmission as a result.

Ingroup reception in the balanced condition was also associated with positive reaction, positive and negative information evaluations, and the evaluation of relevancy. The information received from ingroup neighbors was more likely to be accepted (*OR* = 5.75, 95% CI [4.08, 8.24], *P* = 1.41×10^−22^; Fig 2E), more likely to be positively evaluated (*OR* = 1.23, 95% CI [1.03, 1.45], *P* = 0.019), less likely to be negatively evaluated (*OR* = 0.78, 95% CI [0.65, 0.94], *P* = 0.007; Fig 2G), and more likely to be perceived as relevant to the participants’ social group (*OR* = 1.33, 95% CI [1.11, 1.59], *P* = 0.002). In the ingroup-biased condition, participants were more likely to accept the information received from the ingroup (*OR* = 3.29, 95% CI [1.82, 5.78], *P* = 4.75×10^−5^; Fig 2E), but they consumed less time reviewing it, compared with the outgroup-sent messages (*b =* -0.89, SE_*b*_ = 0.30, *P* = 0.004). See S7 Table for details.

It is worth noting that, while the ingroup-slanted inflow increased the overall frequency of ingroup transmission, each occurrence of ingroup reception had a smaller effect on ingroup transmission in the ingroup-biased condition than in the balanced one. As the negative slope of the ingroup reception in Fig 2B demonstrates, the likelihood of ingroup transmission after ingroup reception decreased by 8 percentage points with the biased inflow. This tendency was also supported by a comparison of ingroup-shared articles sent at the same timepoints in the two conditions (*OR* = 0.65, 95% CI [0.49, 0.84], *P* = 0.001; see S5 Table for details). In sum, the biased inflow reduced the effect of ingroup reception on ingroup transmission, triggering more attempts to escape the closed within-group circulation of information. These increased attempts, however, were not sufficient to change the overall directionality of outflow, which was strongly governed by the biased inflow.

**Fig 3:**
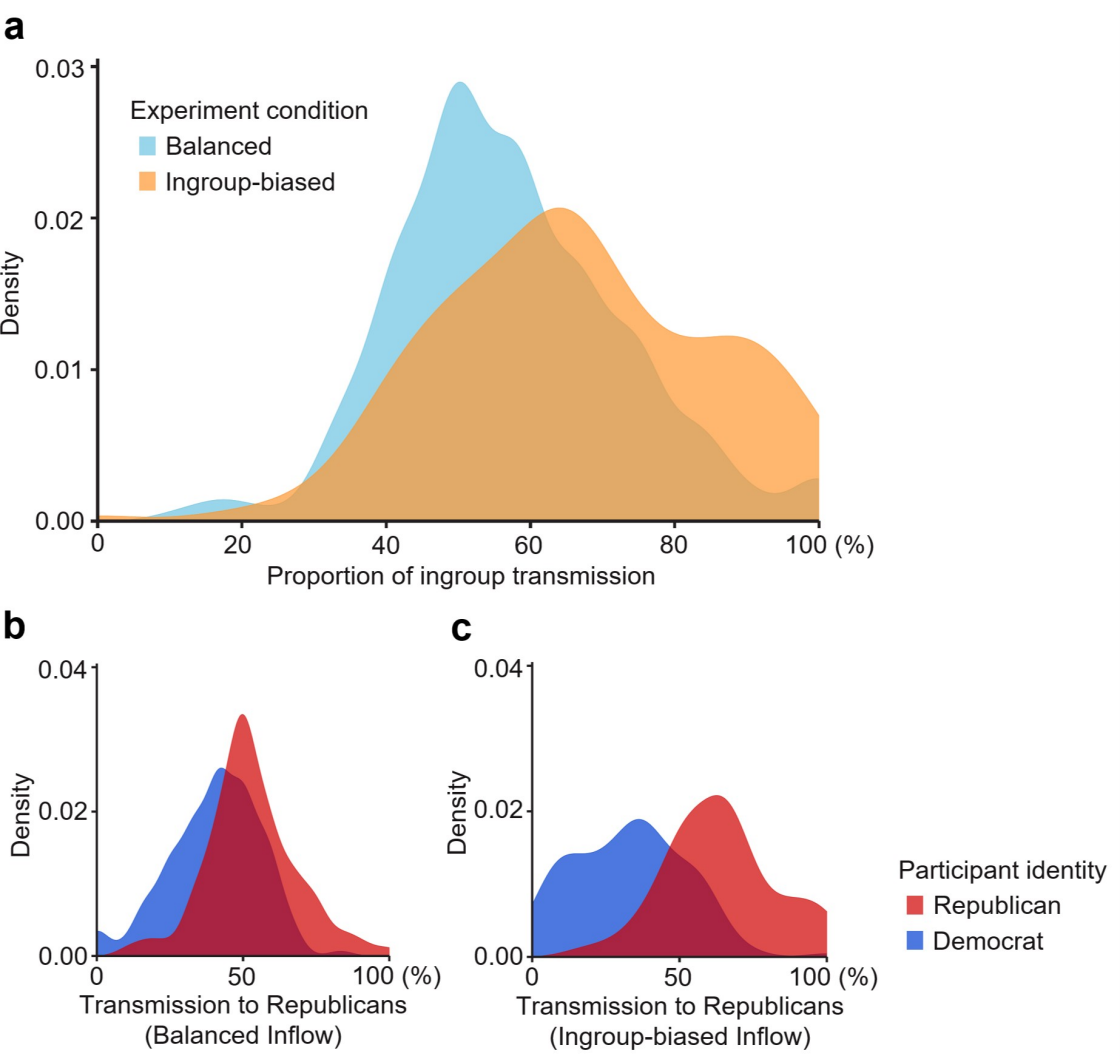
Individual-level effects of the ingroup-biased inflow. **a**. Distribution of the proportion of ingroup transmission. *N* = 432. **b**. Distribution of transmission to Republicans in the balanced condition. *N* = 234. Transmission to Republicans is defined as the proportion of information transmitted to Republican neighbors by a participant. **c**. Distribution of transmission to Republicans in the ingroup-biased condition. *N* = 198.

The effect of the ingroup-slanted inflow was also identified at the individual level, as shown in Fig 3A. I calculated the proportion of ingroup transmission for each participant by dividing the number of ingroup transmissions by the number of all transmissions. The biased inflow increased the proportion of ingroup transmission by 8.75 percentage points (*b =* 8.75, SE_*b*_ = 1.67, *P* = 2.55×10^−7^; see S8 Table for details). The effect size was medium (Cohen’s *d* = 0.52, see Methods for details). This effect was still highly significant within each subgroup: Republicans and Democrats transmitted 11.0% and 7.4% more information, respectively, to their ingroup in the ingroup-biased condition (Republican: *b =* 11.03, SE_*b*_ = 2.71, *P* = 7.19×10^−5^; Democrats: *b =* 7.44, SE_*b*_ = 2.22, *P* = 5.37×10^−4^; S8 Table), generating a polarizing pattern depicted in Fig 3B and 3C.

**Fig 4:**
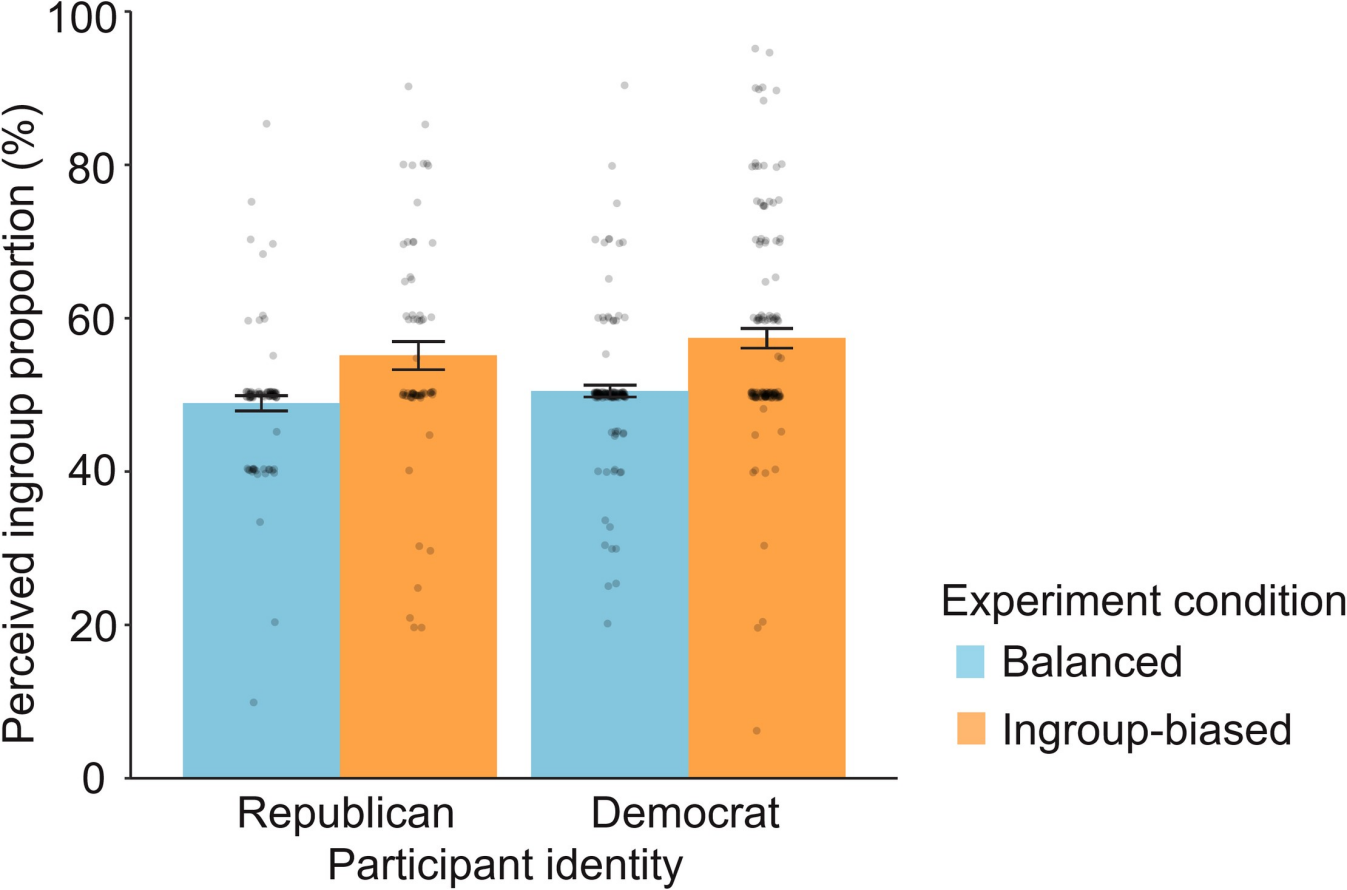
Effect of the ingroup-biased inflow on perceived ingroup proportion. The error bars reflect mean ± s.e.m using bootstrapping. Each point represents an individual response.

Finally, the ingroup-slanted inflow distorted the perception of a majority. As shown in Fig 4, the estimations of ingroup proportion were distributed over a range of values, reflecting participants’ estimation errors, and the ingroup-biased inflow shifted the distribution upward by promoting the misperception that ingroup neighbors are numerically superior in their social networks. Overall, the biased inflow resulted in a 6.63 percentage point increase in the perceived proportion of ingroup neighbors (*b =* 6.63, SE_*b*_ = 1.18, *P* = 3.46×10^−8^; See S9 Table for details). The effect size was medium (*d* = 0.56, see Methods for details). The estimations from Republican and Democrat participants were both increased by 6.2 and 6.9 percentage points, respectively, and these subgroup effects were also highly significant (Republican: *b =* 6.20, SE_*b*_ = 1.96, *P* = 0.002; Democrat: *b =* 6.87, SE_*b*_ = 1.48, *P* = 5.33×10^−6^; S9 Table). Furthermore, the biased inflow caused a false perception of a majority. Both social groups in the ingroup-biased condition significantly overestimated the proportion of ingroup neighbors, compared to the actual value, 50%; that is, they falsely perceived that their own group was the majority on social networks (Republican: M = 55.1, SE = 1.8, two-tailed *t*_(64)_ = 2.76, *P* = 0.008; Democrat: M = 57.4, SE = 1.3, two-tailed *t*_(127)_ = 5.72, *P* = 7.39×10^−8^). On the other hand, participants in the balanced condition correctly detected the numerical balance between the two neighbor groups (M = 49.9, SE = 0.6), and their estimations were not significantly different from the actual value (two-tailed *t*_(226)_ = -0.23, *P* = 0.820).

A separate experiment with 84 participants replicated the main findings of the current experiment. This independent experiment confirmed that ingroup-slanted inflow significantly increases ingroup transmission and perceived ingroup proportion (see Methods for details of this additional experiment).

## Discussion

These findings reveal that ingroup-slanted inflow of information biases the direction of transmission, the social reaction to information exchange, the evaluation of information, and the perception of social networks. First, it was found that ingroup-biased inflow promotes ingroup-biased outflow, which in turn increases the ingroup directionality of inflows of their neighbors. Thus, the finding provides evidence of positive and mutual reinforcement between biased flow of information and biased information behavior. This “spiral of segregation” insulates people from information exchange across group boundaries, generating and maintaining barriers that hinder intergroup communication. Along with informational and structural segregations, this dynamics may also contribute to segregated information flows on social networks [4,19,41], which lead to echo chambers and political polarization.

Second, ingroup-slanted inflow prompts positive reactions to social exchange and suppresses negative evaluations of the exchanged information. Because biased inflows, in this sense, could lead to better social relationships and prevent unpleasant states of arousal, people who are exposed to them may be motivated to maintain and reproduce them. Also, these findings indicate that biased inflows increase the sustainability of ingroup network ties compared with outgroup ones by allowing the within-group channels to mediate more interactions that are socially favorable. Hence, in the long run, the biased inflow may drive the transformation toward more insular social structures.

Third, the experiment demonstrated that ingroup-slanted inflow distorts perceived ingroup proportion on social networks. Combined with the fact that the perception of belonging to the majority encourages people to speak out and to effuse more information [29,30], this result supports a positive reinforcement between biased information flows and the biased perception of social structures, which sheds light on a new aspect of the “spiral of silence” [29,30,45].

The existence of these “spirals” also suggests that a small group of strategic actors may aggravate the separation of information flow within a large group of people. The committed agents on social media, who often make use of artificial agency, are known to transmit information much more frequently and extensively than most users [46,47]. These agents could distort public discourse not only by spreading unsubstantiated content [48,49] but also by exacerbating segregation and polarization through the manipulation of volume and direction of information flows, as shown in this study.

It is also noticeable that the effects of information flow cannot be equated to the mere sum of effects from individual instances of information exchange. The current study demonstrated that, although ingroup-slanted inflow increases the overall frequency of ingroup transmission, it also reduces the likelihood of ingroup transmission following each occurrence of ingroup reception. This means that people are able to recognize an overall imbalance in the information flow and seek to alleviate intergroup segregation by switching the direction of flow from the ingroup to the outgroup. Motives for this “information balancing” behavior may relate to attention captured by rare and infrequent types of social interaction [50,51]. This explanation is supported by the fact that participants in the ingroup-biased condition spent significantly more time consuming a piece of information from outgroup neighbors than that from the ingroup. Future research should explore the mechanism and consequences of this balancing behavior in more detail.

The results also reflect important behavioral patterns of social media users. First, participants discriminated each article based on their social identity and the message content, and the content of an article was associated with its likelihood of ingroup transmission (see Methods for details). This pattern aligns with previous findings that message contents affect information behaviors in social media [11,13,17]. Second, an average participant in the balanced condition sent more information to ingroup neighbors than to the outgroup (see Methods for details). This result reflects ingroup bias, which has been emphasized in previous studies on the social interaction in social media [4,19,41]. These patterns suggest that the present experiment was able to capture important factors influencing realistic information behaviors, and it was possible by accommodating the consideration of social interactions.

The reward feature was one of the elements that enabled this experiment to accommodate social considerations. However, the present results cannot be summarized by a simple strategy for profit maximization. Particularly, one may explain that the participants just followed an optimal strategy that maximizes their profit by sending ingroup-received articles to ingroup members, not considering other factors. Three pieces of evidence may help rule out this hypothesis. First, this hypothesis cannot explain the two behavioral patterns discussed above: the effects of message contents and direct ingroup bias. Second, the present experimental setting did not allow deterministic or probabilistic estimations of the expected outcome of an information exchange, on which a profit maximization strategy could be grounded. This is because a participant’s bonus was supposed to be determined by the judgment of a neighbor whose reward was not affected by the judgment, which makes a rational optimization of profit less feasible. Therefore, a participant in this uncertain situation had to rely on psychological processes, considering how a potential receiver would perceive message contents, and the participant’s identity and sharing behavior. Third, previous studies have identified that social and intrinsic motivations are equally or more important than financial incentives to MTurk participants [52–54]. In this aspect, the alternative hypothesis tends to neglect the important behavioral motives of participants.

The present study suggests several directions for future research. Most of all, the outgroup-biased flow of information may induce very different socio-psychological dynamics than the ingroup-biased flow examined here. Some studies have found that people who perceive a communication environment as biased against their social group attempt to intervene in the situation by correcting some of the information [55,56]. Some scholars argue that increased exposure to information from other groups may even induce backfire effects, exacerbating biased perception and behaviors [57–59]. Also, theories on false consensus explain that people tend to underestimate the prevalence of information supporting their opponents [60,61]. These findings suggest that outgroup-biased flow may trigger distinctive cognitive and behavioral processes, producing outcomes qualitatively different from the present findings. Future work should explore whether and how outgroup-biased flow affects information behavior, information perception, and perceived social networks. Second, researchers should investigate various degrees of ingroup directionality. The present experiment tested an information flow that is more ingroup-slanted than average patterns observed in social media [4,26], and future research should examine how various levels of ingroup directionality influence users and whether there is a “tipping point” triggering much more biased behavior and perception. Third, future study is encouraged to explore in detail why and how ingroup-slanted flow decreases the effect of each ingroup reception on ingroup transmission. Fourth, by comparing experimental results from countries with different characteristics, future research may discover how the level of partisan conflict and the salience of public discourse on polarization influence the present findings. Lastly, scholars have found that other digital platforms, such as instant messaging apps, are also important in understanding the social flow of information [62], and future research could extend the present study to investigate behavior and perception in these platforms.

Defining practically feasible and ecologically valid experimental settings was a challenge for this research. First, although the present study was able to simulate a simple social media environment, the experimental setting tended to emphasize the political dimension of social interactions. Social media users, on the other hand, consume both political and non-political contents, and their behavior and perception are influenced by multiple dimensions of social identity and social status, such as race, gender, and economic status. Thus, social media users may be less responsive to political differences than the participants in the present experiment. Second, implying financial incentives was a first attempt to accommodate social considerations in an experimental setting, but future research should seek to invent more natural ways to accomplish this goal. Third, participants were required to share each article, and they could share it with only one neighbor. While these measures allowed sufficient and detailed observations in an experimental environment, they also restricted the behavior of participants, generating narrower but more frequent information sharing than that in the natural social media environment. Lastly, the consideration of attention span prohibited conducting longer experiments, and the examination of information exchange among human participants was hindered by practical constraints.

Despite these limitations, the present research aimed to investigate the dynamics between social identity, social interaction, and information flow during social propagation. A lack of systematic investigation on these topics has been partly due to the fact that existing studies on information diffusion have been mostly based on observational data [63], while experimental research has not been able to exploit social, dynamic, and interactive experimental designs. To the best of my knowledge, this study is the first one attempting to overcome these limitations. Furthermore, considering the massive scale of social networks, such as Facebook and Twitter, and the enormous amount of information exchanged through these social media every day, the mechanisms identified in the present research may have large societal impacts [64].

## Methods

### Recruitment

A total of 433 participants finished the experiment. Participants were limited to those within the United States. One participant did not open any shared articles and was excluded from the dataset. Therefore, 5,184 observations from 432 participants, 160 Republicans and 272 Democrats, were analyzed. The proportion of the Republican group (160/432 = 37.0%, S12 Table) was within the range of values reported by other MTurk-based studies, such as 39.3% [65], 30.4% [66], 40.3% [67], and 25.6% [68]. 234 participants were assigned to the balanced inflow condition (92 Republicans and 142 Democrats), and 198 participants were assigned to the ingroup-biased inflow condition (68 Republicans and 130 Democrats). The experiment was approved by the Institutional Review Board of the University of Pennsylvania.

MTurk samples are known to be more Democratic and diverse than nationally representative samples [68–70]. However, scholars have demonstrated that experimental results based on MTurk samples can replicate those from nationally representative samples [70–74] and other convenience samples [70,75]. Some studies have found that MTurk produces more reliable results than other means [52,76,77]. Also, MTurk participants were found to mirror the psychological characteristics of the general population including psychological divisions between liberals and conservatives [72,74]. Various studies on social media behaviors [43,44,78] and political behaviors based on cognitive bias [66,79–81] have also utilized MTurk. MTurk was deemed especially appropriate for the present study in which the participants were involved in real-time online social interactions, and MTurk has been widely used for this type of experimental settings [80,82,83]. The analysis of the present study carefully controlled for the differences between political identities in examining the effects of the experimental manipulation on behavioral and cognitive outcomes.

### Participant classification

On the first webpage of the website, participants created a username and provided a worker identification number for compensation. Informed consent was obtained on this page. Participants also answered a question about political identity: “Generally speaking, do you usually think of yourself as a Republican, a Democrat, an Independent, or some other party?”. The participants who chose “Independent” or “Others” in this question were given an additional question about whether they prefer one of the two parties. Based on their answers, all participants were classified into one of the two groups: Democrats and Republicans. (The participants who did not report political preference also entered the experiment, but their responses were excluded from the analysis.)

Participants were informed that their usernames and group membership would be shown to other participants connected to them. The participants were then directed to an instruction page and entered the experiment.

### Experimental interface and procedure

The duration of the experiment was 6 minutes. The experimental interface aimed to accommodate key features of popular social media services, such as Twitter and Facebook, in a controlled environment. First, it included an article list. This list showed articles that were available to each participant, and new articles sent from neighbors were updated to the list in real time. Participants could select an article from the list, review it, and forward it to one of their neighbors. Second, the interface included a neighbor list that displayed all neighbors connected to each participant. Participants could interact with these neighbors by receiving articles from and transmitting articles to those neighbors. The neighbor list showed 12 neighbors, 6 Democrats and 6 Republicans. The order of social identities in the neighbor list was fixed regardless of the identity of participants and experiment conditions (S1 Fig). Each listed neighbor was assigned an avatar and a username which were randomly selected from a set of avatars and a set of usernames, respectively. Neighbor usernames did not contain expressions that could stimulate speculations about a neighbor’s other political characteristics, such as “maga” and “hillary,” and about other social characteristics, such as “white,” and “girl.” The preference for specific positions of the list, such as top positions, was not identified, as shown in S10 Table.

Participants received 12 articles from their neighbors. These shared articles were sequentially added to the top of the article list. For each shared article, a username and a social identity of a neighbor who shared the article were displayed in the article list. When a new article was received, a notification indicating that a neighbor “shared a post” was shown in the neighbor list for 30 seconds. In addition, 2 base articles were given to participants by default. These articles aimed at providing a more realistic experimental environment and did not include sender information. Responses to the base articles were excluded from the analysis.

Each experimental condition corresponds to an ingroup/outgroup identity sequence of sending neighbors. In the balanced inflow condition, the sequence was IN, OUT, OUT, IN, IN, IN, OUT, OUT, IN, OUT, IN, and OUT (IN: ingroup neighbor, OUT: outgroup neighbor). The sequence for the ingroup-biased inflow condition was IN, IN, IN, IN, IN, OUT, IN, IN, IN, IN, IN, and IN. (Randomizing the sequences of sending neighbor identities and estimating an average treatment effect over various sequences would increase the external validity of the findings. However, it would necessitate a larger sample due to a greater variation in the treatment effect. Thus, given limited resources available to the study, these sequences were fixed in the experiment.) Article update timings were identical in both conditions.

The experiment started with the reception phase. When participants selected an article, a popup appeared. The popup, which overrode the article list, contained the title and the content of the selected article, a 30-second timer displaying remaining reading time, an identity and a username of the sending neighbor, and two buttons for social reaction. After reading the article, the participants answered a question, “Do you accept [the sender’s username]'s sharing?”, by clicking on one of the two buttons: “Accept” and “Reject.” The base articles included only a “Next” button. These buttons were activated 5 seconds after opening an article. Clicking a reaction button advanced the popup to the transmission phase. If participants did not click a button, the popup automatically proceeded to the transmission phase upon an expiration of the reading timer. In the transmission phase, participants selected one neighbor from the neighbor list to forward the selected article. Participants were informed that “5¢ will be added to your winnings if the neighbor you choose accepts your sharing. Otherwise, 5¢ will be deducted from your winnings.” Once participants chose a neighbor, they could close the popup by clicking a “Close” button and return to the reception phase. Hence, participants were expected to repeat a set of behaviors during the experiment: choosing and reading an article and sharing it with a neighbor. Participants were required to transmit each article that he/she had selected, and opening the same article multiple times was not allowed. A debriefing page followed the post-experiment questionnaire.

On the recruiting post on MTurk, participants were informed that $1.10 was guaranteed for a 10-minute experiment. This rate of payment is comparable to those of other studies using the same recruitment platform [82,84]. Because each reaction from a neighbor was expected to result in an aforementioned penalty or bonus, the participants would expect that these incentives could accumulate up to ±63.6% of the guaranteed credit. However, since the neighbors were, in effect, preprogrammed agents, the actual and final amount of payment shown after the experiment did not reflect the incentives during the experiment: the final payment was calculated only based on the number of articles a participant had selected, as explained on the debriefing page, and it ranged between $1.10 and $1.18.

The experiments were conducted in January and February 2018, and in July and August 2018. Due to a technical reason, participants recruited during the second period received additional 1¢ upon the completion of their participation. The two periods did not yield statistically different outcomes.

#### Articles

42 articles describing current social and political topics of the United States were quoted from reports published by Pew Research Center in 2017 (available on www.pewresearch.org) and were adjusted in length (the average number of paragraphs = 2.69, SD = 0.68; the average number of characters in an article = 1046, SD = 139). Titles of the articles used in the experiment are shown in S1 Table. These reports are expected to take relatively neutral viewpoints, compared to news reports or opinion editorials from traditional media outlets [26,85,86]. The content of the articles was based on survey results and government data, and the topics included gun control, immigration, race and ethnicity, and American politics. 12 shared articles and 2 base articles were randomly chosen for a participant among the 42 articles.

#### Measurement

For each article sent from a neighbor, I recorded ingroup reception (a binary variable indicating whether an article was received from an ingroup neighbor), positive reaction (a binary variable indicating whether sharing of an article was accepted), and ingroup transmission (a binary variable indicating whether an article was transmitted to an ingroup neighbor). Reading time (time spent reading an article) was measured in seconds. Positive reaction, ingroup transmission, and reading time were recorded as missing for articles which were not selected by a participant. Three questions about information evaluation followed the experiment. In the first question, participants were given a list of titles they had selected during the experiment and chose articles which were supporting opinions of their social group. For articles not chosen by a participant, the variable was recorded as missing. In the same manner, the second and the third questions asked about articles which were challenging and relevant to opinions of their social group, respectively. These questions were followed by a question measuring the perceived ingroup proportion: “In the previous session, what percentage of your neighbors were of your political group?”. Numerical answers for this question ranged from 0 to 100.

### Descriptive statistics

Basic behavioral statistics were compared between the two conditions using a t-test adjusting for correlation within a social group [87]. The difference in the number of selected articles between the two conditions (*Z* = 0.13, *P* = 0.897), the difference in the average reading time of a participant in the two conditions (*Z* = 0.37, *P* = 0.715), and the difference in the number of selected unique neighbors between the two conditions (*Z* = 1.90, *P* = 0.058) were not statistically significant.

### Statistical methods for behavior-level analysis

The behavior-level analyses were based on random effect logistic regression models which included random effects of participants (*N* = 5,184 nested in 432 participants). As a robustness check, I also tested other statistical models handling clustering, and all methods generated nearly identical results, as S3 Table shows. The behavioral-level analysis of ingroup transmission for articles received from the ingroup was based on a random effect logistic regression model which included random effects of participants. This analysis used records on 5 articles sent by ingroup neighbors at the same timepoints in both experiment conditions (*N* = 2,160 nested in 432 participants). The individual-level analysis of the proportion of ingroup transmission was conducted using an ordinary linear regression model (*N* = 432). The individual-level analysis of the perceived proportion of ingroup neighbors was based on an ordinary linear regression model (*N* = 432).

### Effect size

Effect sizes were examined at the individual level based on Cohen’s *d* [88]. For the effect of the ingroup-biased inflow on the proportion of ingroup transmission, effect sizes were 0.52 (all participants), 0.65 (Republican subgroup), and 0.43 (Democrat subgroup). For the effect of the ingroup-biased inflow on the perceived proportion of the ingroup, effect sizes were 0.56 (all participants), 0.52 (Republican subgroup), and 0.57 (Democrat subgroup). The effect sizes of the ingroup-biased inflow on positive social reaction and on negative evaluation were 0.18 for both outcomes, when considering all participants.

### Content effect

The articles shown in the experiment were more positively evaluated by Democrat participants than Republicans. In the balanced condition, Democrat participants were significantly more likely to perceive articles as positive (*OR* = 1.61, 95% CI: [1.25, 2.08], *P* = 2.65×10^−4^) and relevant (*OR* = 1.38, 95% CI: [1.05, 1.83], *P* = 0.020), adjusting for the effect of ingroup sender identity (S7 Table). Also in the ingroup-biased condition, Democrat participants were significantly more likely to evaluate the articles as positive (*OR* = 1.47, 95% CI: [1.11, 1.95], *P* = 0.007) and relevant (*OR* = 1.51, 95% CI: [1.05, 2.18], *P* = 0.024), and significantly less likely to perceive negativity (*OR* = 0.65, 95% CI: [0.49, 0.84], *P* = 0.001), adjusting for the effect of ingroup sender identity (S7 Table).

The evaluations of each article were associated with its likelihood of ingroup transmission. As shown in S11 Table, the associations between ingroup transmission and the perceptions of positivity, negativity, and relevancy were highly significant. To be specific, in the balanced condition, participants were more likely to transmit a message to an ingroup neighbor when the message was favorable and relevant to their social group, and messages negatively evaluated by participants were less likely to be sent to the ingroup (positive evaluation: *OR* = 1.81, 95% CI: [1.53, 2.14], *P* = 7.32×10^−12^; negative evaluation: *OR* = 0.57, 95% CI: [0.47, 0.67], *P* = 1.10×10^−10^; relevancy evaluation: *OR* = 1.48, 95% CI: [1.24, 1.76], *P* = 1.05×10^−5^). These associations were consistent in the ingroup-biased condition (positive evaluation: *OR* = 1.84, 95% CI: [1.50, 2.26], *P* = 6.03×10^−9^; negative evaluation: *OR* = 0.50, 95% CI: [0.40, 0.61], *P* = 1.18×10^−10^; relevancy evaluation: *OR* = 1.51, 95% CI: [1.22, 1.87], *P* = 1.50×10^−4^).

An article-level analysis also confirms the behavioral patterns shown above. In this analysis, the responses of participants were aggregated for each article. The analysis demonstrated that a social group discriminated congruent and incongruent information, and the content of an article was correlated with its ingroup transmission probability. As shown in S2A–S2C Fig, Democrat and Republican participants discriminated articles based on their social group. To be specific, the two groups made opposite evaluations on the same article: as Republicans evaluated an article as more positive and relevant, Democrats evaluated it as less positive and relevant (positive evaluation: *r*_(40)_ = -0.626, *P* = 9.14×10^−6^; relevancy evaluation: *r*_(40)_ = -0.451, *P* = 0.003; S2A and S2B Fig). Also, articles that were perceived as more negative by Republican participants were perceived as less negative by Democrats (*r*_(40)_ = -0.473, *P* = 0.002; S2C Fig). Furthermore, there was a strong association between positive perception and ingroup transmission of an article, as shown in S2D and S2E Fig. Specifically, within each social group of participants, information that was more favorable to their social identity was more likely to be sent to their ingroup neighbors (Democrat: *r*_(40)_ = 0.606, *P* = 2.10×10^−5^; Republican: *r*_(40)_ = 0.572, *P* = 7.48×10^−5^). These pieces of evidence showed that participants identified and discriminated articles based on their social identity, and the content of each article influenced their transmission behaviors. The consistent patterns with high levels of statistical significance are especially impressive given that these correlations were calculated using only 42 articles.

S13 Table provides more extensive evidence supporting that participants identified and discriminated each article based on their social identity. Within each social group, the positive evaluation and the negative evaluation of an article were negatively correlated, and the positive evaluation and the relevancy evaluation of an article were positively correlated. Also, within each group of participants, the positivity and the relevancy of an article were positively correlated with its likelihood of ingroup transmission, while the negativity of an article was negatively correlated with the likelihood. See S13 Table for details. These results confirm that participants evaluated and discriminated each article based on their social identity.

### Direct ingroup bias

The results demonstrated direct ingroup bias of participants: they were more likely to interact with ingroup neighbors. Participants in the control condition sent more information to their ingroup members on average, although they received the same amount of information from each group of neighbors. Specifically, the estimated probability of ingroup transmission averaged over the two social groups was 56.2%, and it was significantly greater than 50% (95% CI: [54.2%, 58.2%], the estimation was based on the model specified in S6 Table). This result showed that participants preferred to interact with and expected more positive reactions from ingroup members.

### Replication

A separate experiment was conducted in January 2018 and replicated the main findings of the current study. Experimental procedures were identical to the current experiment, except that participants were recruited separately for each condition. 37 participants (21 Democrats and 16 Republicans) were recruited for the balanced inflow condition, and 47 participants (32 Democrats and 15 Republicans) were recruited for the ingroup-biased inflow condition. This experiment produced 1008 behavioral records nested in 84 participants, consisting of 444 observations nested in 37 participants of the balanced condition and 564 observations nested in 47 participants of the biased condition.

## Supporting information

S1 Fig Example screenshots of the experimental interface. (DOCX)

S2 Fig Information perceptions and transmission behaviors in the balanced inflow condition aggregated at the article level. (DOCX)

S1 Table Titles of the articles used in the experiment. (DOCX)

S2 Table Random effects logistic regression models predicting ingroup transmission. (DOCX)

S3 Table Comparison of estimates from different statistical methods handling clustering. (DOCX)

S4 Table Random effects regression models predicting information behaviors and perceptions. (DOCX)

S5 Table Random effects logistic regression model predicting ingroup transmission for information sent from the ingroup. (DOCX)

S6 Table Random effects logistic regression models predicting ingroup transmission in the balanced inflow condition. (DOCX)

S7 Table Random effects regression models predicting information behaviors and perceptions in each experiment condition. (DOCX)

S8 Table Ordinary linear regression models predicting the percentage of ingroup transmission. (DOCX)

S9 Table Ordinary linear regression models predicting the perceived percentage of ingroup neighbors. (DOCX)

S10 Table Frequency of selection by position in the neighbor list. (DOCX)

S11 Table Association between information perceptions and the transmission behavior. (DOCX)

S12 Table Social identity of participants. (DOCX)

S13 Table Correlation between article-level responses from republican and democrat participants in the balanced condition. (DOCX)
