# Supplementary material for: Directionality of information flow and echoes without chambers
Source: PLoS One. 2019 May 15;14(5):e0215949. doi: 10.1371/journal.pone.0215949 (PMC6519792; doi:10.1371/journal.pone.0215949)
Supplement: S1 Fig — (DOCX) [file pone.0215949.s001.docx]

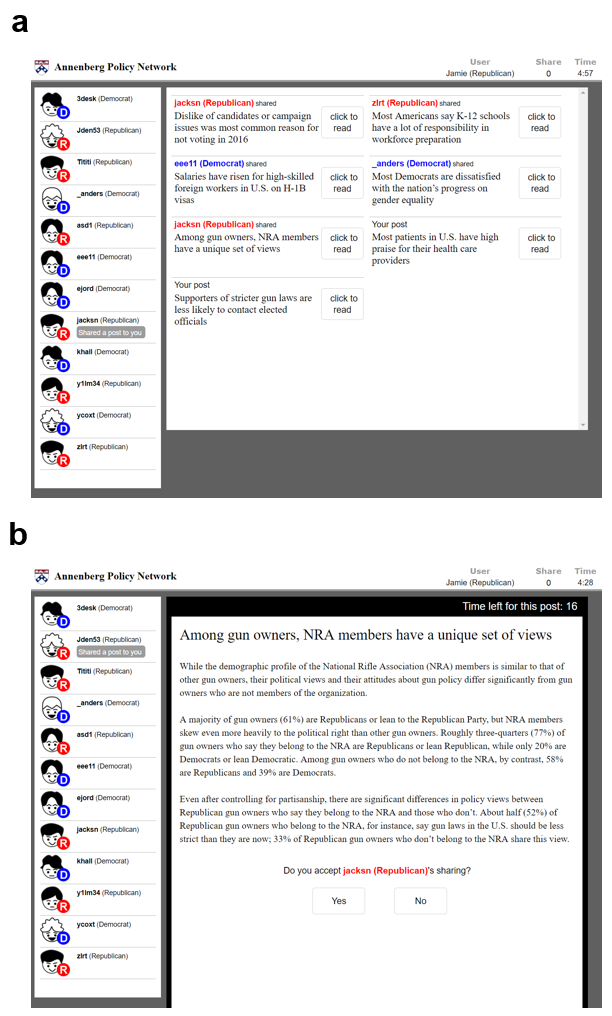


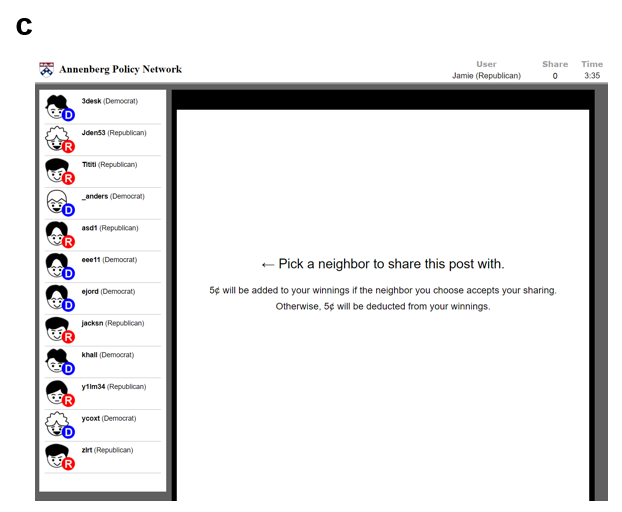


**S1 Fig. Example Screenshots of the Experimental Interface**. These screenshots illustrate a case of a Republican participant. **a**. The reception phase with the article list. **b**. The reception phase after selecting an article. **c**. The transmission phase.
