# Supplementary material for: Directionality of information flow and echoes without chambers
Source: PLoS One. 2019 May 15;14(5):e0215949. doi: 10.1371/journal.pone.0215949 (PMC6519792; doi:10.1371/journal.pone.0215949)
Supplement: S2 Fig — (DOCX) [file pone.0215949.s002.docx]

***
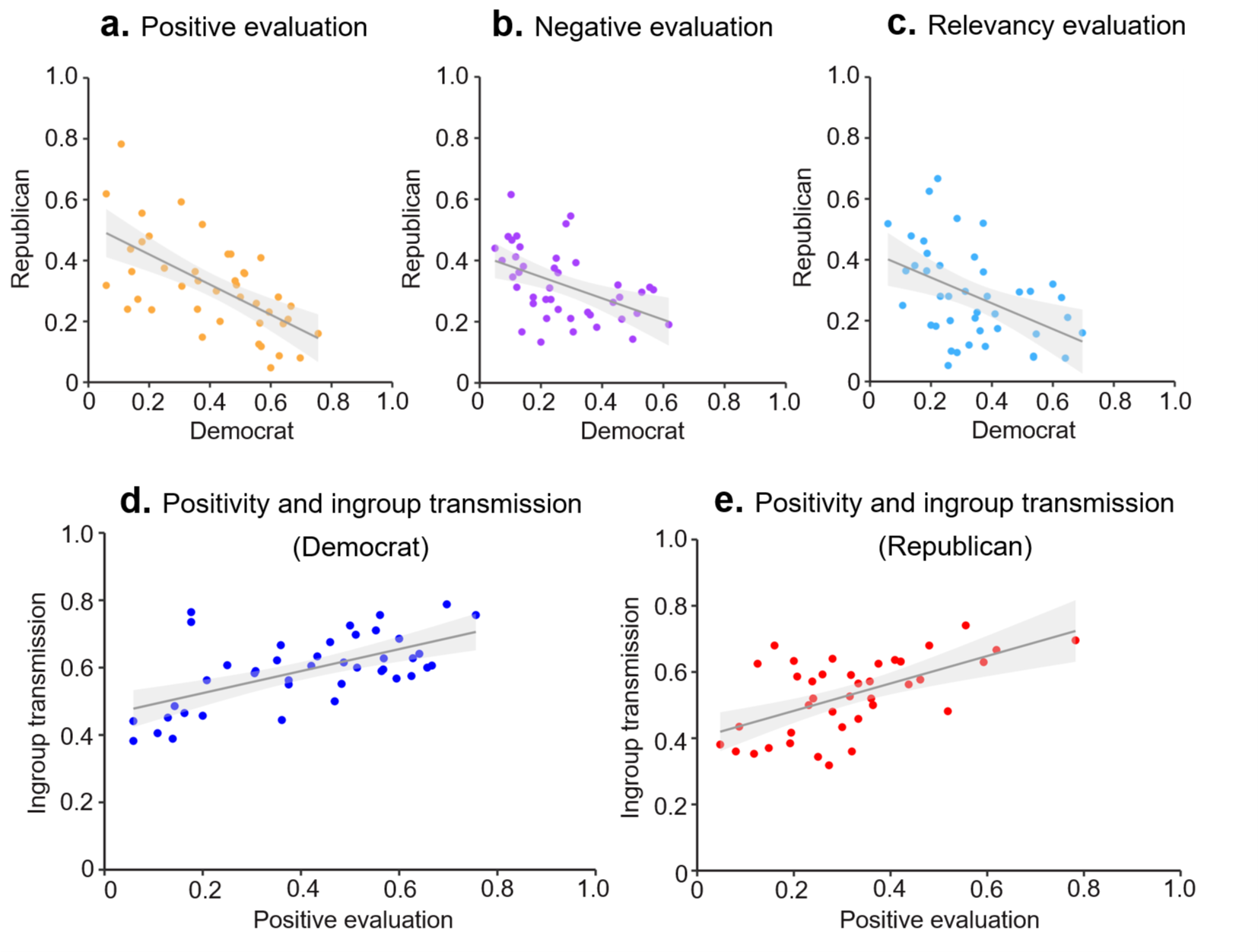
***

**S2 Fig. Information Perceptions and Transmission Behaviors in the Balanced Inflow Condition Aggregated at the Article Level.** Each plot contains 42 dots, and each dot represents an article. The values of positive evaluation, negative evaluation, and relevancy evaluation were the proportions of Democrat [Republican] participants in the balanced inflow condition who reported an article as positive, negative, and relevant, respectively, among those who selected the article. These measures ranged between 0 to 1. Ingroup transmission was the proportion of Democrat [Republican] participants in the balanced inflow condition who transmitted an article to an ingroup neighbor among those who selected the article. Ingroup transmission ranged between 0 and 1. Each grey line represents the slope of a regression line calculated from a linear regression model based on the two variables in each plot. A grey area represents the standard error of a slope. **a.** Correlation between positive evaluations among Republican and among Democrat participants (r_(40)_ = -0.626, P = 9.14×10^-6^). **b.** Correlation between negative evaluations among Republican and among Democrat participants (r_(40)_ = -0.473, P = 0.002). **c.** Correlation between relevancy evaluations among Republican and among Democrat participants (r_(40)_ = -0.451, P = 0.003). **d.** Correlation between positive evaluation and ingroup transmission among Democrat participants (r_(40)_ = 0.606, P = 2.10×10^-5^). **e.** Correlation between positive evaluation and ingroup transmission among Republican participants (r_(40)_ = 0.572, P = 7.48×10^-5^).
