# Supplementary material for: Directionality of information flow and echoes without chambers
Source: PLoS One. 2019 May 15;14(5):e0215949. doi: 10.1371/journal.pone.0215949 (PMC6519792; doi:10.1371/journal.pone.0215949)
Supplement: S1 Table — (DOCX) [file pone.0215949.s003.docx]

**S1 Table. Titles of the Articles Used in the Experiment.**

|  | Index | Title | Index | Title |  |
| --- | --- | --- | --- | --- | --- |
|  | 1 | Americans are split on the principle of pre-emptive military force | 22 | On abortion, persistent divides between - and within - the two parties |  |
|  | 2 | Among gun owners, NRA members have a unique set of views | 23 | One-in-seven U.S. infants were multiracial or multiethnic |  |
|  | 3 | Among Republican gun owners, women are more supportive of restricting gun ownership | 24 | One million immigrants receive lawful permanent resident status each year |  |
|  | 4 | Bipartisan support for some gun proposals, stark partisan divisions on many others | 25 | Public support for 'single payer' health coverage grows |  |
|  | 5 | Black voter turnout fell in 2016, even as a record number of Americans cast ballots | 26 | Republicans are skeptical about colleges' impact on U.S. |  |
|  | 6 | Democratic voters are increasingly likely to call their views liberal | 27 | Republicans much “colder” than Democrats in views of professors |  |
|  | 7 | Dislike of candidates or campaign issues was most common reason for not voting in 2016 | 28 | Republicans’ optimism about future of GOP declines |  |
|  | 8 | Few Americans support cuts to most government programs, including Medicaid | 29 | Rural and urban gun owners have different experiences, views on gun policy |  |
|  | 9 | For election news, young people turned to some national papers more than their elders | 30 | Salaries have risen for high-skilled foreign workers in U.S. on H-1B visas |  |
|  | 10 | Globally, more people see U.S. power and influence as a major threat | 31 | Supporters of stricter gun laws are less likely to contact elected officials |  |
|  | 11 | In global popularity contest, U.S. and China vie for first | 32 | The federal government’s total debt stands at $19.845 trillion |  |
|  | 12 | Majorities in Europe, North America worried about Islamic extremism | 33 | The U.S. Asian population grew 72% between 2000 and 2015 |  |
|  | 13 | Many minority students go to schools where at least half of their peers are their race or ethnicity | 34 | U.S. active-duty military presence overseas is at its smallest in decades |  |
|  | 14 | More Millennial households are in poverty than households headed by any other generation | 35 | U.S. Hispanic population growth has leveled off |  |
|  | 15 | More than half of new green cards go to people already living in the U.S. | 36 | U.S. House seats rarely flip to other party in special elections |  |
|  | 16 | Most Americans say K-12 schools have a lot of responsibility in workforce preparation | 37 | Views about whether whites benefit from societal advantages split along racial and partisan lines |  |
|  | 17 | Most Americans unaware that as U.S. manufacturing jobs have disappeared, output has grown | 38 | Views of racism as a major problem increase sharply, especially among Democrats |  |
|  | 18 | Most Americans view openness to foreigners as essential to who we are as a nation | 39 | Views toward Muslims are more positive than they were in 2014 |  |
|  | 19 | Most Democrats are dissatisfied with the nation’s progress on gender equality | 40 | Whites and blacks differ widely in their views on police officers |  |
|  | 20 | Most Democrats say a person’s gender can be different from the sex they were assigned at birth | 41 | Women’s views of nation’s prospects take a negative turn |  |
|  | 21 | Most patients in U.S. have high praise for their health care providers | 42 | 1 in 4 black Americans have faced online harassment because of their race or ethnicity |  |
| *Note*. The titles are listed in alphabetical order. | | | | | |
