# Supplementary material for: Directionality of information flow and echoes without chambers
Source: PLoS One. 2019 May 15;14(5):e0215949. doi: 10.1371/journal.pone.0215949 (PMC6519792; doi:10.1371/journal.pone.0215949)
Supplement: S2 Table — (DOCX) [file pone.0215949.s004.docx]

**S2 Table. Random Effects Logistic Regression Models Predicting Ingroup Transmission.**

| Predictor | Participant identity | | |
| --- | --- | --- | --- |
|  | All | Republican | Democrat |
| Ingroup-biased inflow | 1.45 ***  [1.27, 1.67] | 1.55 ***  [1.24, 1.94] | 1.40 ***  [1.17, 1.67] |
| Democrat participant | 1.26 **  [1.09, 1.46] |  |  |
| Constant | 1.16 *  [1.02, 1.31] | 1.13  [0.98, 1.30] | 1.49 ***  [1.32, 1.68] |
| *Notes*. **P* < 0.05, ***P* < 0.01, ****P* < 0.001. Estimates are odd ratios. All: *N* = 5,184 observations nested in 432 participants. Republican: *N* = 1,920 observations nested in 160 participants, Democrat: *N* = 3,264 observations nested in 272 participants. Listwise deletion was used to handle missing data. | | | |
