# Supplementary material for: Directionality of information flow and echoes without chambers
Source: PLoS One. 2019 May 15;14(5):e0215949. doi: 10.1371/journal.pone.0215949 (PMC6519792; doi:10.1371/journal.pone.0215949)
Supplement: S3 Table — (DOCX) [file pone.0215949.s005.docx]

**S3 Table. Comparison of Estimates from Different Statistical Methods Handling Clustering.**

| Predictor | Random Effects | GEE | Cluster VCOV |
| --- | --- | --- | --- |
| Ingroup-biased inflow | 0.37 ***  (0.07) | 0.36 ***  (0.07) | 0.36 ***  (0.07) |
| Democrat participant | 0.23 **  (0.07) | 0.23 **  (0.07) | 0.23 **  (0.07) |
| Constant | 0.15 *  (0.06) | 0.14 *  (0.06) | 0.14 *  (0.06) |
| *Note*. All models predict ingroup transmission. **P* < 0.05, ***P* < 0.01, ****P* < 0.001. *N*=5,184 observations nested in 432 participants. Estimates are regression coefficients. Listwise deletion was used to handle missing data. For the GEE (generalized estimating equations^1^) logistic regression model, a working correlation matrix with exchangeable structure was estimated. The model with Cluster VCOV (multi-way cluster-robust variance-covariance matrix estimators^2^) considered clustering by participants. The heteroskedasticity-consistent covariance matrix estimators^3^ were used for GEE and Cluster VCOV.  ^1^ Liang K-Y, Zeger SL. Longitudinal data analysis using generalized linear models. Biometrika. 1986;73: 13–22.  ^2^ Cameron AC, Gelbach JB, Miller DL. Robust Inference With Multiway Clustering. J Bus Econ Stat. 2011;29: 238–249. doi:10.1198/jbes.2010.07136  ^3^ White H. A Heteroskedasticity-Consistent Covariance Matrix Estimator and a Direct Test for Heteroskedasticity. Econometrica. 1980;48: 817. doi:10.2307/1912934 | | | |
