# Supplementary material for: Directionality of information flow and echoes without chambers
Source: PLoS One. 2019 May 15;14(5):e0215949. doi: 10.1371/journal.pone.0215949 (PMC6519792; doi:10.1371/journal.pone.0215949)
Supplement: S4 Table — (DOCX) [file pone.0215949.s006.docx]

**S4 Table. Random Effects Regression Models Predicting Information Behaviors and Perceptions.**

|  |  | Predictor | Ingroup  transmission^1^ | | Positive reaction^1^ | | Reading time ^2^ | | Positive evaluation^1^ | Negative evaluation^1^ | | Relevancy evaluation^1^ | |  | |  |
| --- | --- | --- | --- | --- | --- | --- | --- | --- | --- | --- | --- | --- | --- | --- | --- | --- |
|  |  | Ingroup-biased inflow | | 1.45 ***  [1.27, 1.67] | | 2.06 **  [1.26, 3.42] | | -0.40  (0.41) | 1.07  [0.89, 1.28] | | 0.83 *  [0.69, 0.99] | | 1.02  [0.82, 1.26] | |  | |
|  |  | Democrat participant | | 1.26 **  [1.09, 1.46] | | 1.30  [0.78, 2.14] | | 1.03 *  (0.43) | 1.54 ***  [1.28, 1.87] | | 0.72 ***  [0.60, 0.86] | | 1.44 **  [1.15, 1.80] | |  | |
|  |  | Constant | | 1.16 *  [1.02, 1.31] | | 24.16 ***  [15.12, 41.79] | | 10.41 ***  (0.38) | 0.43 ***  [0.37, 0.51] | | 0.46 ***  [0.39, 0.54] | | 0.34 ***  [0.28, 0.41] | |  | |
|  | *Note.* **P* < 0.05, ***P* < 0.01, ****P* < 0.001. *N* = 5,184 observations nested in 432 participants. Listwise deletion was used to handle missing data.  ^1^ A random effects logistic regression model. Estimates are odd ratios. 95% confidence intervals in brackets.  ^2^ A random effects linear regression model. Reading time was measured in seconds. Estimates are regression coefficients of the model. Standard errors in parentheses. | | | | | | | | | | | | | | |  |
