# Supplementary material for: Directionality of information flow and echoes without chambers
Source: PLoS One. 2019 May 15;14(5):e0215949. doi: 10.1371/journal.pone.0215949 (PMC6519792; doi:10.1371/journal.pone.0215949)
Supplement: S5 Table — (DOCX) [file pone.0215949.s007.docx]

**S5 Table. Random Effects Logistic Regression Model Predicting Ingroup Transmission for Information Sent from the Ingroup.**

| Predictor | Odd Ratio |
| --- | --- |
| Ingroup-biased inflow | 0.65 **  [0.49, 0.84] |
| Democrat participant | 1.44 **  [1.10, 1.89] |
| Intercept | 3.04 ***  [2.39, 3.92] |
| *Note. N* = 2,160 observations nested in 432 participants. These observations are records of 5 articles (1^st^, 4^th^, 5^th^, 9^th^, 11^th^), which were sent from ingroup members at the same timepoints in both experiment conditions. 95% confidence intervals in brackets. ***P* < 0.01, ****P* < 0.001. Listwise deletion was used to handle missing data. | |
