# Supplementary material for: Directionality of information flow and echoes without chambers
Source: PLoS One. 2019 May 15;14(5):e0215949. doi: 10.1371/journal.pone.0215949 (PMC6519792; doi:10.1371/journal.pone.0215949)
Supplement: S6 Table — (DOCX) [file pone.0215949.s008.docx]

**S6 Table. Random Effects Logistic Regression Models Predicting Ingroup Transmission in the Balanced Inflow Condition.**

| Predictor | Participant identity | | |
| --- | --- | --- | --- |
|  | All | Republican | Democrat |
| Ingroup reception | 5.56 ***  [4.67, 6.65] | 5.36 ***  [4.08, 7.10] | 5.71 ***  [4.55, 7.20] |
| Democrat participant | 1.37 **  [1.13, 1.67] |  |  |
| Intercept | 0.47 ***  [0.40, 0.56] | 0.48 ***  [0.39, 0.59] | 0.64 ***  [0.55, 0.75] |
| *Notes*. ***P* < 0.01, ****P* < 0.001. Estimates are odd ratios. All: *N* = 2,808 observations nested in 234 participants. Republican: *N* = 1,104 observations nested in 92 participants, Democrat: *N* = 1,704 observations nested in 142 participants. Listwise deletion was used to handle missing data. | | | |
