# Supplementary material for: Directionality of information flow and echoes without chambers
Source: PLoS One. 2019 May 15;14(5):e0215949. doi: 10.1371/journal.pone.0215949 (PMC6519792; doi:10.1371/journal.pone.0215949)
Supplement: S7 Table — (DOCX) [file pone.0215949.s009.docx]

**S7 Table. Random Effects Regression Models Predicting Information Behaviors and Perceptions in Each Experiment Condition.**

|  |  | Balanced inflow | | | | | | |  | |
| --- | --- | --- | --- | --- | --- | --- | --- | --- | --- | --- |
|  |  | Predictor | Ingroup  transmission^1^ | Positive reaction^1^ | Reading time ^2^ | Positive evaluation^1^ | Negative evaluation^1^ | Relevancy evaluation^1^ |  | |
|  |  | Ingroup reception | 5.56 ***  [4.67, 6.65] | 5.75 ***  [4.08, 8.24] | -0.05  (0.18) | 1.23 *  [1.03, 1.45] | 0.78 **  [0.65, 0.94] | 1.33 **  [1.11, 1.59] |  | |
|  |  | Democrat  participant | 1.37 **  [1.13, 1.67] | 1.09  [0.54, 2.18] | 1.42 *  (0.59) | 1.61 ***  [1.25, 2.08] | 0.78  [0.60, 1.01] | 1.38 *  [1.05, 1.83] |  | |
|  |  | Intercept | 0.47 ***  [0.40, 0.56] | 15.40 ***  [8.58, 31.41] | 10.20 ***  (0.47) | 0.38 ***  [0.30, 0.47] | 0.49 ***  [0.39, 0.61] | 0.30 ***  [0.24, 0.38] |  | |
|  |  | Ingroup-biased inflow | | | | | | |  | |
|  |  | Predictor | Ingroup  transmission^1^ | Positive reaction^1^ | Reading time ^2^ | Positive evaluation^1^ | Negative evaluation^1^ | Relevancy evaluation^1^ |  | |
|  |  | Ingroup reception | 4.70 ***  [3.38, 6.58] | 3.29 ***  [1.82, 5.78] | -0.89 **  (0.30) | 1.33  [0.96, 1.86] | 0.79  [0.56, 1.12] | 1.24  [0.87, 1.78] |  | |
|  |  | Democrat  participant | 1.20  [0.92, 1.58] | 1.71  [0.72, 4.17] | 0.55  (0.61) | 1.47 **  [1.11, 1.95] | 0.65 **  [0.49, 0.84] | 1.51 *  [1.05, 2.18] |  | |
|  |  | Intercept | 0.44 ***  [0.30, 0.64] | 18.12 ***  [7.55, 54.26] | 11.14 ***  (0.57) | 0.37 ***  [0.25, 0.54] | 0.51 ***  [0.35, 0.74] | 0.27 ***  [0.17, 0.41] |  | |
|  | *Note.* **P* < 0.05, ***P* < 0.01, ****P* < 0.001. Balanced: *N* = 2,808 observations nested in 234 participants. Ingroup-biased: *N* = 2,376 observations nested in 198 participants. Listwise deletion was used to handle missing data.  ^1^. A random effects logistic regression model. Estimates are odd ratios. 95% confidence intervals in brackets.  ^2^. A random effects linear regression model. Reading time was measured in seconds. Estimates are regression coefficients of the model. Standard errors in parentheses. | | | | | | | | |  |
