# Supplementary material for: Directionality of information flow and echoes without chambers
Source: PLoS One. 2019 May 15;14(5):e0215949. doi: 10.1371/journal.pone.0215949 (PMC6519792; doi:10.1371/journal.pone.0215949)
Supplement: S8 Table — (DOCX) [file pone.0215949.s010.docx]

**S8 Table. Ordinary Linear Regression Models Predicting the Percentage of Ingroup Transmission.**

| Predictor | Participant identity | | |
| --- | --- | --- | --- |
|  | All | Republican | Democrat |
| Ingroup-biased inflow | 8.75 ***  (1.67) | 11.03 ***  (2.71) | 7.44 ***  (2.12) |
| Democrat participant | 5.23 **  (1.72) |  |  |
| Constant | 53.88 ***  (1.54) | 52.91 ***  (1.76) | 59.74 ***  (1.47) |
| *Notes*. ***P* < 0.01, ****P* < 0.001. All: *N* = 432, Republican: *N* = 160, Democrat: *N* = 272. Standard errors in parentheses. Listwise deletion was used to handle missing data. | | | |
