# Supplementary material for: Directionality of information flow and echoes without chambers
Source: PLoS One. 2019 May 15;14(5):e0215949. doi: 10.1371/journal.pone.0215949 (PMC6519792; doi:10.1371/journal.pone.0215949)
Supplement: S9 Table — (DOCX) [file pone.0215949.s011.docx]

**S9 Table. Ordinary Linear Regression Models Predicting the Perceived Percentage of Ingroup Neighbors.**

| Predictor | Participant identity | | |
| --- | --- | --- | --- |
|  | All | Republican | Democrat |
| Ingroup-biased inflow | 6.63 ***  (1.18) | 6.20 **  (1.96) | 6.87 ***  (1.48) |
| Democrat participant | 1.89  (1.22) |  |  |
| Constant | 48.71 ***  (1.09) | 48.89 ***  (1.27) | 50.49 ***  (1.03) |
| *Notes*. ***P* < 0.01, ****P* < 0.001. All: *N* = 432, Republican: *N* = 160, Democrat: *N* = 272. Standard errors in parentheses. Listwise deletion was used to handle missing data. | | | |
