# Supplementary material for: Directionality of information flow and echoes without chambers
Source: PLoS One. 2019 May 15;14(5):e0215949. doi: 10.1371/journal.pone.0215949 (PMC6519792; doi:10.1371/journal.pone.0215949)
Supplement: S10 Table — (DOCX) [file pone.0215949.s012.docx]

**S10 Table. Frequency of Selection by Position in the Neighbor List.**

|  | Positions in the neighbor list | Neighbor identity | Proportion (%) |  |
| --- | --- | --- | --- | --- |
|  | 1^st^ (Top) | D | 7.9 |  |
|  | 2^nd^ | R | 6.4 |  |
|  | 3^rd^ | R | 5.8 |  |
|  | 4^th^ | D | 8.2 |  |
|  | 5^th^ | R | 9.9 |  |
|  | 6^th^ | D | 8.1 |  |
|  | 7^th^ | D | 10.7 |  |
|  | 8^th^ | R | 8.3 |  |
|  | 9^th^ | D | 9.8 |  |
|  | 10^th^ | R | 7.8 |  |
|  | 11^th^ | D | 10.9 |  |
|  | 12^th^ (Bottom) | R | 6.2 |  |
|  | Total |  | 100.0 |  |
| *Note*. D: Democrat, R: Republican. *N* = 4,758 observations. | | | | |
