# Supplementary material for: Directionality of information flow and echoes without chambers
Source: PLoS One. 2019 May 15;14(5):e0215949. doi: 10.1371/journal.pone.0215949 (PMC6519792; doi:10.1371/journal.pone.0215949)
Supplement: S11 Table — (DOCX) [file pone.0215949.s013.docx]

**S11 Table. Association Between Information Perceptions and the Transmission Behavior**

|  | Balanced inflow | | | |  |
| --- | --- | --- | --- | --- | --- |
|  | Information perception | Positive evaluation | Negative evaluation | Relevancy evaluation |  |
|  | Association with ingroup transmission  (Odd ratio) | 1.81 ***  [1.53, 2.14] | 0.57 ***  [0.48, 0.67] | 1.48 ***  [1.24, 1.76] |  |
|  |  |  |  |  |  |
|  |  |  |  |  |  |
|  | Ingroup-biased inflow | | | |  |
|  | Information perception | Positive evaluation | Negative evaluation | Relevancy evaluation |  |
|  | Association with ingroup transmission  (Odd ratio) | 1.84 ***  [1.50, 2.26] | 0.50 ***  [0.40, 0.61] | 1.51 ***  [1.22, 1.87] |  |
|  |  |  |  |  |  |
|  |  |  |  |  |  |
| *Note.* ****P* < 0.001. Balanced: *N* = 2,808 observations nested in 234 participants. Ingroup-biased: *N* = 2,376 observations nested in 198 participants. The association between an information perception variable and ingroup transmission was measured by the regression coefficient of the information perception variable in a random effects logistic regression model fitted to ingroup transmission, adjusting for sending neighbor identity and participant identity. Estimates are odd ratios. 95% confidence intervals in brackets. Listwise deletion was used to handle missing data. | | | | | |
