# Supplementary material for: Directionality of information flow and echoes without chambers
Source: PLoS One. 2019 May 15;14(5):e0215949. doi: 10.1371/journal.pone.0215949 (PMC6519792; doi:10.1371/journal.pone.0215949)
Supplement: S12 Table — (DOCX) [file pone.0215949.s014.docx]

**S12 Table. Social Identity of Participants**

|  | Category | Self-identification | Control  (Balanced) | Treatment  (Ingroup-biased) | Total |  |
| --- | --- | --- | --- | --- | --- | --- |
|  | Republican group | Republican | 68 | 56 | 124 |  |
|  |  | Lean to Republican | 24 | 12 | 36 |  |
|  |  | Republican group total | 92 | 68 | 160 |  |
|  | Democratic group | Democrat | 112 | 106 | 218 |  |
|  |  | Lean to Democrat | 30 | 24 | 54 |  |
|  |  | Democrat group total | 142 | 130 | 272 |  |
|  | **Total** | | **234** | **198** | **432** |  |
| *Note*: Lean to Republican [Democrat] refers to a group of participants who did not identify themselves as a Republican [a Democrat] but identified themselves as closer to the Republican party [the Democratic party]. There were 64 respondents who did not identify themselves as a Republican or a Democrat and did not report themselves as closer to one of the two parties. These respondents were not included in the table and were also excluded from the analysis. | | | | | | |
