# Supplementary material for: Directionality of information flow and echoes without chambers
Source: PLoS One. 2019 May 15;14(5):e0215949. doi: 10.1371/journal.pone.0215949 (PMC6519792; doi:10.1371/journal.pone.0215949)
Supplement: S13 Table — (DOCX) [file pone.0215949.s015.docx]

**S13 Table. Correlation Between Article-level Responses from Republican and Democrat Participants in the Balanced Condition.**

|  | | Response from Republicans | | | | Response from Democrats | | | |  |
| --- | --- | --- | --- | --- | --- | --- | --- | --- | --- | --- |
|  |  | Positive | Negative | Relevant | Ingroup | Positive | Negative | Relevant | Ingroup |  |
| Response from Republicans | Positive evaluation |  | -0.310  * | 0.672  *** | 0.572  *** | -0.626 *** | 0.684  *** | -0.507  *** | -0.434 ** |  |
|  | Negative evaluation |  |  | -0.241 | -0.343  * | 0.587  *** | -0.473 ** | 0.521  *** | 0.347  * |  |
|  | Relevant evaluation |  |  |  | 0.391  * | -0.585  *** | 0.607  *** | -0.451 ** | -0.404 ** |  |
|  | Ingroup transmission |  |  |  |  | -0.320  * | 0.513  *** | -0.239 | -0.104 |  |
|  |  |  |  | Response from Democrats | Positive evaluation |  | -0.850 *** | 0.776 *** | 0.606 *** |  |
|  |  |  |  |  | Negative evaluation |  |  | -0.604 *** | -0.509  *** |  |
|  |  |  |  |  | Relevant evaluation |  |  |  | 0.424  ** |  |
|  |  |  |  |  | Ingroup transmission |  |  |  |  |  |
| *Note.* **P* < 0.05, ***P* < 0.01, ****P* < 0.001. *N* = 42 articles. Each value in this table represents a correlation. The positive evaluation, negative evaluation, and relevancy evaluation of Republicans were the proportion of Republican participants who reported an article as positive, negative, and relevant, respectively, among those who selected the article. These measures ranged between 0 and 1. Ingroup transmission of Republicans was the proportion of Republican participants who transmitted an article to the ingroup among those who selected the article. It ranged between 0 and 1. Similarly, the four variables were calculated for Democrat participants. | | | | | | | | | | |
